# Pressure‐Induced Broadband Emission of 2D Organic–Inorganic Hybrid Perovskite (C_6_H_5_C_2_H_4_NH_3_)_2_PbBr_4_

**DOI:** 10.1002/advs.201801628

**Published:** 2018-11-24

**Authors:** Long Zhang, Lianwei Wu, Kai Wang, Bo Zou

**Affiliations:** ^1^ State Key Laboratory of Superhard Materials College of Physics Jilin University Changchun 130012 China

**Keywords:** amorphization, band‐gap narrowing, broadband emission, perovskites, pressure

## Abstract

2D Ruddlesden–Popper halide perovskites, which incorporate hydrophobic organic interlayers to considerably improve environmental stability and optical properties diversity, have attracted substantial research attention for optoelectronic applications. The burgeoning broad emission arising from exciton self‐trapping of 2D perovskites shows a strong dependence on a deformable structure. Here, the pressure‐induced broadband emission of layered (001) Pb‐Br perovskite with a large Stokes shift in the visible region is observed by finely improving lattice distortion to increase exciton–phonon coupling under hydrostatic pressure. Band gap narrows ≈0.5 eV under modest pressure, mainly due to the large compressibility of the orientational organic layer, confirming that the bulky organic cations notably influence the structure and, in turn, the various properties of materials. Sequential amorphization of the organic and inorganic layer is confirmed by high pressure Raman and X‐ray diffraction measurements, suggesting the particularity of layered crystal structures. The mechanism constructed here offers a new route for tuning the optical properties of 2D perovskites.

The emerging Ruddlesden–Popper 2D metal halide perovskites have received considerable attention and are frequently employed in photovoltaic or optoelectronic device owing to their superior environmental stability and excellent tunability.^1^ 2D organic–inorganic metal halide perovskites possess a layered sandwich structure composed of alternating organic and inorganic components, giving rise to remarkable quantum confinement effects and large exciton binding energy, in which the generated carries are confined within the inorganic layers. This multiple quantum‐well structures can form energy funnels, leading to the more efficient radiative recombination for light‐emitting diode (LED) application.^2^ The bulky phenyl ethyl‐ammonium (PEA) organic molecule spacer cation is applied to synthesize 2D or quasi‐2D Ruddlesden–Popper lead bromide perovskites, which are used in photovoltaic solar cells with higher open circuit voltage and stability compared to the 3D perovskite solar cells.^3^ The LEDs based on (C_6_H_5_C_2_H_4_NH_3_)_2_PbBr_4_ [(PEA)_2_PbBr_4_] exhibit strong room‐temperature color‐pure violet emission with a narrow bandwidth originating from the natural quantum confinement effects, which is rare in inorganic or organic LEDs.^4^ Importantly, these devices exhibit long‐term stability and lifetime against light and moisture due to incorporated hydrophobic PEA^+^ cations, implicating a bright application prospect for this material.^5^

Recently, broadband emission from 2D Pb‐Br or Pb‐Cl perovskite without extrinsic emissive sites inspired the emergence of the optoelectronic research field; it exhibits potential applications in artificial illumination and displays to substitute inorganic phosphors with a complex preparation process; such potential stems from exciton self‐trapping associated with strong exciton–phonon coupling.^6^ The stable self‐trapped excitons in a deformable lattice can result in a highly distorted excited state with respect to the ground state. The structure–property relationship studies of a series of 2D hybrid perovskites with various bulky organic cations indicating the self‐trapped exciton emission are closely related to the distorted inorganic sublattice. At ambient conditions, the broadband emission of (PEA)_2_PbCl_4_ was observed to originate from self‐trapped excitons in a highly distorted structure.^7^ On the contrary, (PEA)_2_PbBr_4_ with relatively smaller distorted lattice only exhibits narrow free exciton emission. Given this huge difference in emission, the broad emission of (PEA)_2_PbBr_4_ may be activated by continuously tuning the structural distortion to improve exciton–phonon coupling with the assistance of high‐pressure technology at room temperature, thereby confirming the relation between broad emission and structure. As a powerful and clean tool, hydrostatic pressure can effectively tune the crystal strucutre and electronic landscapes of 2D perovskite materials without chemical interference; it uniquely accessed novel photophysical and transport properties not accessible by traditional ways, providing a perfect way to confirm our conjecture.^8^ In recent years, high pressure research of halide perovskites mainly focused on 3D structure, realizing the fine tuning of optical and electronic properties by lattice compression.^9^ Unprecedented simultaneous band‐gap narrowing and carrier lifetime prolongation of FAPbI_3_ were achieved by modifying the bond length and bond angle under mild pressure.^10^ The electronic conductivity of halide perovskite can be greatly enhanced with compression. In particular, band‐gap closure and semiconductor‐to‐metal transition of MAPbI_3_ imply a whole new electronic configuration and transport properties.^11^ Strikingly, the partial retainability of high‐pressure superior properties of pressure‐treated materials upon decompression will encourage scientists to synthesize more excellent photovoltaic materials at ambient condition, which is vitally significant for practical applications.^12^

Herein, we report the evolution of optical properties of 2D Ruddlesden–Popper metal halide perovskite (PEA)_2_PbBr_4_ by applying hydrostatic pressure up to ≈28 GPa in a controlled manner, the highest pressure applied in this study. The pressure‐driven crystal structure transitions together with optoelectronic properties response were characterized by in situ high‐pressure photoluminescence (PL), UV–vis absorption, synchrotron X‐ray diffraction (XRD), and Raman experiments. Our studies have opened up opportunities for fundamental research between local structure and electronic properties, and practical applications on a new route of functional optoelectronic and photovoltaic devices based on 2D Ruddlesden–Popper metal halide perovskites.

The pressure response of the photovoltaic‐related optical properties is of utmost concern. Thus, we first carried out in situ high‐pressure PL experiments. **Figure** **1**a shows the collected PL spectra of (PEA)_2_PbBr_4_ upon compression. At ambient conditions, we observed a sharp and narrow violet free exciton emission centered at 416 nm with a lower energy PL tail originates from radiative recombination of trap states due to the remarkable quantum and dielectric confinement effects in layer 2D perovskites.[[qv: 4a,13]] As the pressure increased, the initial narrow emission (NE) underwent a gradual redshift with the decrease in PL intensity. We noticed a sizable drop in PL intensity of the NE at ≈4 GPa. A weak lower‐energy emission appeared, showing a broad red emission (BE) spanning the 500–900 nm region with a very large Stokes shift (Figure S2, Supporting Information). This phenomenon confirms our prediction. With the further increase in pressure, the significant increase in BE is accompanied by continuous weakening of NE. Upon compression to ≈8.0 GPa, the NE completely disappeared, whereas the emission intensity of BE reached its maximum. Then, the BE gradually weakened and finally disappeared at 15.6 GPa. The PL micrograph shows the PL intensity and color changes under high pressure (illustration in Figure 1, 4a). The distinct mixed color of blue and red implies the presence of two emission peaks at 5.9 GPa. The pure BE exhibited a bright red emission, as evidenced by the PL micrograph at 7.7 GPa.

**Figure 1:**
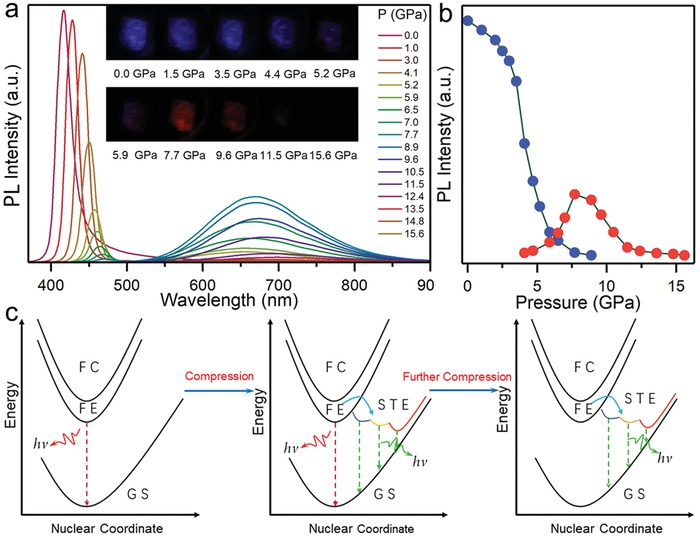
Emission property and broadband emission mechanism. a) PL spectra of (PEA)_2_PbBr_4_ as a function of pressure at room temperature. The illustrations are PL micrographs upon compression. b) Pressure‐induced PL intensity evolution of (PEA)_2_PbBr_4_. c) Schematic illustrations of emission evolutions upon compression. Ground state (GS), free‐carrier state (FC), free‐exciton state (FE), and various self‐trapped exciton state (STE).

Recently, the broadband emission in partial 2D layered perovskites has been discovered and attended as a burgeoning field of study under ambient conditions.^14^ The exciton self‐trapping associated with inorganic lattice distortions and strong exciton–phonon coupling cause broad emission with a large Stokes shift. Figure 1c shows the emission evolution mechanism of (PEA)_2_PbBr_4_ under pressure. At ambient conditions, the photogenerated carriers cannot form stable self‐trapped excitons in an insufficient deformable lattice, which rapidly detrap from the self‐trapped exciton states back to a free‐carrier state before the self‐trapped exciton emission pathway. Therefore, only the narrow emission of free exciton is detected. With the increase in lattice distortion upon compression, strong exciton–phonon coupling in a deformed lattice leads to carrier localization, indicating the formation of stable self‐trapped excitons with multiple self‐trapped states. On the other hand, the self‐trapped exciton state decreasingly detraps back into the free‐exciton state, as reflected by the dramatic decrease of free exciton emission accompanied by broad emission emergence at ≈4 GPa (Figure 1b). An energy distribution of self‐trapped exciton states with respect to the ground state broadens the emission.[[qv: 6d]] With the further increase in pressure, the BE emission intensity reached the peak value along with the disappearance of NE emission, suggesting that the gradually enhanced exciton–phonon coupling hindered self‐trapped excitons returned to the free‐exciton state in a serious deformable lattice. We noted that the pressure suppression effect of PL also contributed to the disappearance of NE emission. Eventually, the BE emission decreased gradually and completely disappeared at ≈16 GPa, coinciding with the appearance of progressive amorphization, which implies the existence of nonradiative recombination in the amorphous lattice based on previous reports.^15^ The distortion of the inorganic layer and lattice amorphization are exemplified by synchrotron XRD results, as well as discussed later.

The modulation process of band gap was traced by UV–vis absorption spectroscopy upon compression of up to 28.4 GPa (**Figure** **2**a). The ambient sample showed a steep exciton absorption edge located at ≈419 nm corresponding to electronic transitions from predominantly Pb(6s)‐Br(4p) mixed states to Pb(6p) states in the inorganic layer.^16^ As the pressure increased, the absorption edge showed a pronounced redshift before ≈12 GPa, followed by a notable blueshift until ≈15 GPa. Then, a slow redshift re‐emerged under higher pressure range. Figure 2b shows the piezochromic transitions of crystals upon compression, from initial colorless transparency to translucent yellow (≈12 GPa) and then turn to translucent light yellow at 14.2 GPa. Under higher pressures, the crystal color gradually deepened again, coincident with the evolution of the absorption spectrum. The band gap of the material was estimated by extrapolating the linear portion of (*αdhν*)^2^ versus the *hν* curve (illustration in Figure 2c), where α is the absorption coefficient, *d* is the sample thickness, and *hν* is the photon energy. At ambient pressure, the band gap was estimated to be 2.96 eV, coincident with previous study results.[[qv: 4a]] We achieved an unprecedented band‐gap narrowing up to ≈0.5 eV in the first redshift process (<12 GPa), which is of great significance for (PEA)_2_PbBr_4_, as doped photovoltaic materials are applied to the perovskite solar cells and may considerably improve photovoltaic device performance, implying prominent band‐gap modifiability in 2D halide perovskite by lattice compression.

**Figure 2:**
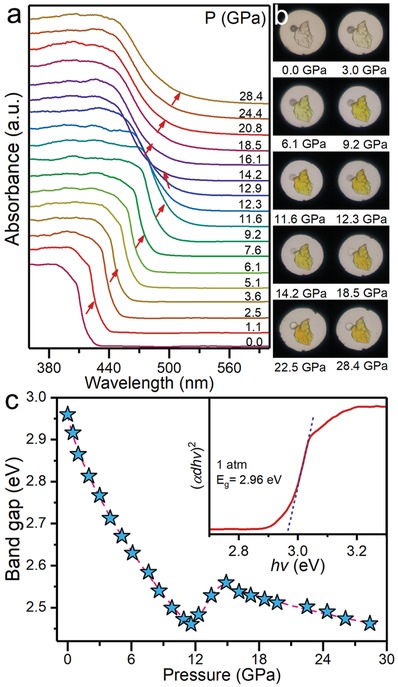
Absorption properties and band‐gap evolution. a) Absorption spectra of (PEA)_2_PbBr_4_ upon compression. b) Piezochromic transitions of (PEA)_2_PbBr_4_ crystals in the DAC chamber. c) Band gap of (PEA)_2_PbBr_4_ as a function of pressure calculated from Tauc plots. The illustration shows the band‐gap Tauc plots at 1 atm.

Remarkable optical property change in (PEA)_2_PbBr_4_ is derived from lattice compression. Therefore, we performed in situ high‐pressure powder XRD experiments at room temperature to track the crystal structure evolution. **Figure** [qv: **3**]a displays the integrated XRD profiles upon compression of up to 21.9 GPa. With the increase in pressure, all Bragg diffraction peaks shifted continuously along the large 2θ direction due to lattice contraction, and no new XRD peaks were observed. Upon compression above ≈12 GPa, considerable degree of pressure‐induced structural distortion appeared, as evidenced by the disappearance of several original reflections in the diffraction patterns and anisotropic peak broadening and weakening, suggesting the beginning of structural amorphization. As the applied pressure further increased to ≈22 GPa, the disappearance of all Bragg diffraction peaks and only a weak broad band suggest the amorphous phase of the material. We performed Rietveld refinement to index the crystal structure of (PEA)_2_PbBr_4_ under ambient conditions (Figure 3d). Our refinement date indicates that the 2D (PEA)_2_PbBr_4_ crystallizes in the triclinic space group *P*‐1with lattice *a* = 11.61 Å, *b* = 11.62 Å, and *c* = 17.57 Å, consistent with previously reported results.^17^ Figure 3b shows the layered crystal structure of (PEA)_2_PbBr_4_ along the crystallographic *c‐*axis. The single inorganic layer was constructed by corner‐sharing PbBr_6_ octahedra alternate with asymmetric organic PEA^+^ cation bilayers. The amino group of bulky organic cations linked the Pb‐Br inorganic framework by two different hydrogen bonds, including bonding to bridging and terminal Br atoms. The weak van der Waals interaction in adjacent PEA^+^ organic layers and the inorganic framework was distorted for accommodating bulky organic molecules (Figure 3c). This hierarchical crystal structure and the existence of various interactions suggest anisotropic lattice pressure responses upon compression. Upon compression, all Pb‐Br‐Pb angles (along the *a* and *b‐*axes) in the inorganic layer gradually reduced due to the tilting and rotation of PbBr_6_ octahedra relative to its neighboring octahedra along the *c‐*axis. Meanwhile, the bridging Br atoms progressively deviated from the *ab* plane (Figures S3 and S4, Supporting Information). Thus, the net result presents greater deformation in the Pb‐Br inorganic framework.

**Figure 3:**
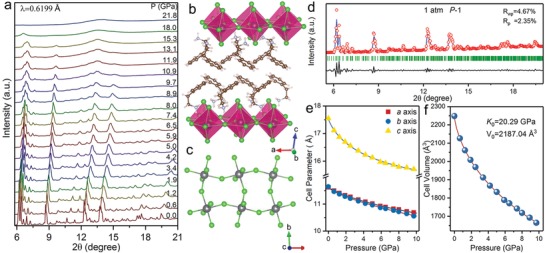
XRD patterns and lattice parameters. a) Representative powder X‐ray diffraction patterns of (PEA)_2_PbBr_4_ upon compression up to 21.8 GPa at room temperature. b) Crystal structure of (PEA)_2_PbBr_4_ at ambient conditions, viewed along the *b‐*axis. Gray, green, brown, violet, and pink spheres represent Pb, Br, C, N, and H atoms, respectively. c) Distorted Pb‐Br inorganic framework layer at ambient condition, viewed along the *c*‐axis. d) Rietveld refinement result of (PEA)_2_PbBr_4_ at ambient conditions. e) Pressure dependence of lattice constants (PEA)_2_PbBr_4_ as a function of pressure. f) Cell volume evolutions of (PEA)_2_PbBr_4_ upon compression.

Figure 3f shows the variations in lattice constants and cell volume. The triclinic (PEA)_2_PbBr_4_ material exhibited distinct anisotropic axial compressibility. The compressibility of the *c‐*axis is far greater than that of *a* and *b‐*axes upon compression, corresponding to the sandwich structure composed of alternating accumulated organic and inorganic layers. In comparison with the low compressible inorganic framework sublattices, the bendable alkylammonium link in the bulky organic cations exhibits greater space flexibility to achieve high compressibility. The second‐order Birch–Murnaghan equation of state was used to fit the pressure‐volume relationships. The fitting bulk modulus (*K*_0_) of (PEA)_2_PbBr_4_ is 20.29 GPa, which is far less than those of inorganic oxide perovskites that usually possess *K*_0_ > 100 GPa.^18^ The large compressibility implies that compression/strain can be a very effective potential way to tune the crystal structure, which is desirable.

To investigate the behavior of the organic layer and its interplay with the inorganic sublattice layer, we carried out high‐pressure Raman experiments at room temperature to provide direct information about the PEA^+^ cation changes. The low‐frequency Raman vibrational modes (50–200 cm^−1^) are associated with the Pb‐Br inorganic layer (**Figure** **4**). On the contrary, the vibrational spectra over the 200–1700 cm^−1^ range are attributable to the PEA^+^ organic cations. With the increase in pressure, Pb‐Br vibrational modes showed evident blueshift, ascribed to the lattice contraction. Upon compression to 7.6 GPa, broad weak vibrational bands appeared in the low‐frequency range. Simultaneously, the high‐frequency vibrational bands maintained well‐defined spectral features, indicating that the inorganic layer is first distorted to provide enough space for accommodating bulky organic molecules. At the applied pressure of up to 11.7 GPa, the vibrational modes of Pb‐Br inorganic sublattice completely disappeared, accompanied by the disappearance and broadening of some high‐frequency vibrational modes, suggesting that seriously distorted inorganic layers cannot meet the flexible space needs for organic cations. Therefore, the bulky PEA^+^ organic cations gradually distorted under higher pressure. The novel sequential distortion mechanism under compression has never been observed in previous 3D or 2D halide perovskite studies.^19^ Upon decompression, the amorphous material restored to its original state, in agreement with the PL and absorption measurement (Figures S5 and S6, Supporting Information). The structural memory effect is attributed to flexible bulky organic cations, which can act as templates for the recovery process.^20^

**Figure 4:**
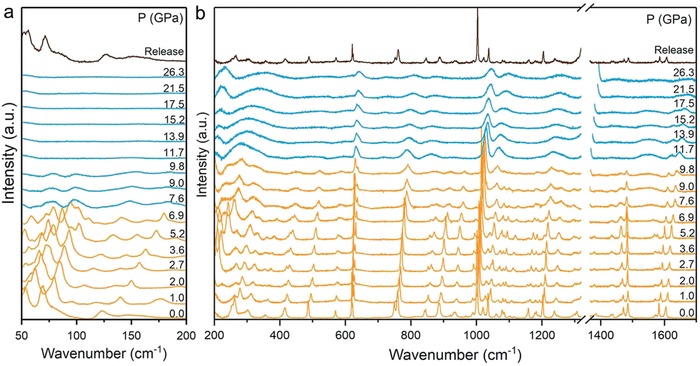
Raman spectra of (PEA)_2_PbBr_4_ as a function of pressure. a) Low‐frequency range and b) high‐frequency range.

Two distinct mechanisms of band‐gap narrowing coincided with the unique structural evolution behavior upon compression through our structural analysis. The dramatic band‐gap narrowing in the low‐pressure range interrelates with the orientated soft organic cation layer.^21^ With the notable layer‐to‐layer compression, the enhancement orbital overlap of Pb s and Br p states pushes the valence band maximum as the electronic band dispersion increases.[[qv: 15b]] On the contrary, the conduction band minimum is mainly a nonbonding localized state of Pb p orbitals, which are insensitive to lattice compression or pressure, therefore, resulting in a decreased band gap. The competition of compression effect with interlayer bond contraction and lattice distortion causes moderate band‐gap narrowing in the high‐pressure range. The destruction of long‐range order due to severely deformed crystal structure will decrease metal halide orbital overlap and electronic band dispersion. Therefore, the partial contribution of interlayer bond contraction to the band‐gap narrowing was counteracted. The pressured‐induced “blue jump” of the band gap at ≈12 GPa is mainly attributed to the distortion of Pb‐Br inorganic layer with less compressibility that decreases metal halide orbital coupling.[[qv: 12b]]

In summary, our results illustrate the strong structure dependence and pressure effect of the optical properties of 2D Ruddlesden–Popper perovskite. The broad emission of (PEA)_2_PbBr_4_ originating from exciton self‐trapping was successfully confirmed to enhance exciton–phonon coupling by increasing lattice distortion. The anomalous band‐gap evolution is ascribed to sequential distortion of organic and inorganic layer due to the large difference in compressibility. Our study results not only elaborate the relationship between broad emission and deformable inorganic sublattice but also map a promising route to access new broadband emission perovskite materials and improve photovoltaic performance by designing a reasonable chemical synthesis under practical conditions.

## Conflict of Interest

The authors declare no conflict of interest.

## Supporting information

Supplementary
