# Supplementary material for: Pressure‐Induced Broadband Emission of 2D Organic–Inorganic Hybrid Perovskite (C6H5C2H4NH3)2PbBr4
Source: Adv Sci (Weinh). 2018 Nov 24;6(2):1801628. doi: 10.1002/advs.201801628 (PMC6343061; doi:10.1002/advs.201801628)
Supplement: Supplementary file 1 — Supplementary [file ADVS-6-1801628-s001.pdf]

Copyright WILEY-VCH Verlag GmbH & Co. KGaA, 69469 Weinheim, Germany, 2018.

ADVANCED SCIENCE
Open Access

Supporting Information

for *Adv. Sci.,* DOI: 10.1002/advs.201801628

Pressure-Induced Broadband Emission of 2D Organic–Inorganic Hybrid Perovskite $(C_6H_5C_2H_4NH_3)_2PbBr_4$

Long Zhang, Lianwei Wu, Kai Wang, and Bo Zou**

Copyright WILEY-VCH Verlag GmbH & Co. KGaA, 69469 Weinheim, Germany, 2016.

Supporting Information

Pressure-Induced Broadband Emission of 2D Organic-Inorganic Hybrid Perovskite $(C_6H_5C_2H_4NH_3)_2PbBr_4$

Long Zhang, Lianwei Wu, Kai Wang,* and Bo Zou*

Experimental Section

Sample preparation and high-pressure generation

The sample of $(PEA)_2PbBr_4$ was purchased from Xi'an Polymer Light Technology Corp. A symmetrical 400 μm culet diamond anvil cell (DAC) was employed to generate high pressure condition. A pre-indented T301 stainless steel gasket with 45 μm thickness has a 150 μm hole as a sample chamber. A small ruby ball was loaded into the sample chamber to determine pressure according to the ruby fluorescence technique. Silicone oil and argon were used as the pressure-transmitting medium (PTM).

In situ high-pressure PL, absorption and Raman measurements

In situ high-pressure PL measurements were carried out by a 355 nm line of a UV DPSS laser with the power of 10 mW. In situ high-pressure UV-Vis absorption measurements were performed by a deuterium-halogen light source. The fiber spectrometer is an Ocean Optics QE65000 spectrometer. In situ High-pressure Raman measurements were carried out by using a 785 nm diode laser with the power of 10 mW and a Raman spectrometer (iHR 550, Symphony II, Horiba Jobin Yvon).

In situ high-pressure XRD measurements

In situ high-pressure angle-dispersive XRD measurements with a wavelength of 0.6199 Å beam were performed at the 4W2 High Pressure Station in Beijing Synchrotron Radiation

Facility and repeated at the BL15U1 beamline of the Shanghai Synchrotron Radiation Facility. The diffraction patterns were integrated into one-dimensional profile by using Fit2D program. The Reflex module combined in Materials Studio was used for Rietveld refinement.

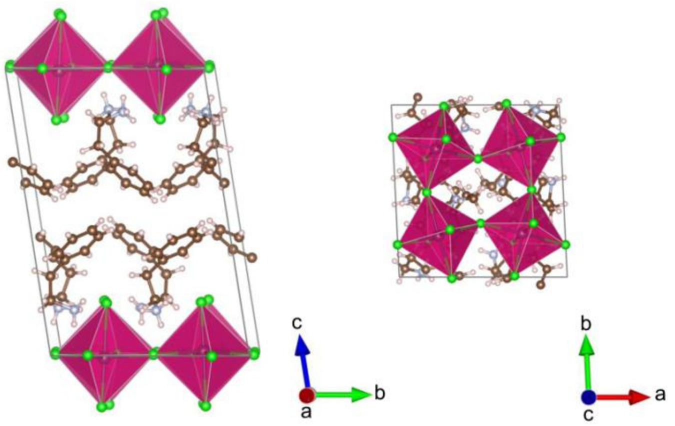

Figure S1. The crystal structure of $(PEA)_2PbBr_4$ at ambient conditions.

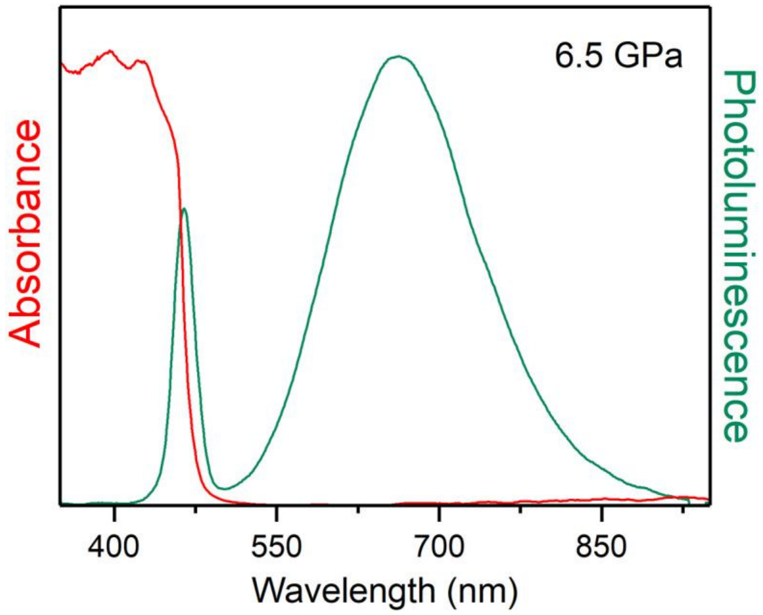

Figure S2. Absorption (red) and PL (green) spectra of $(PEA)_2PbBr_4$ at 6.5 GPa, showing a large Stokes shift.

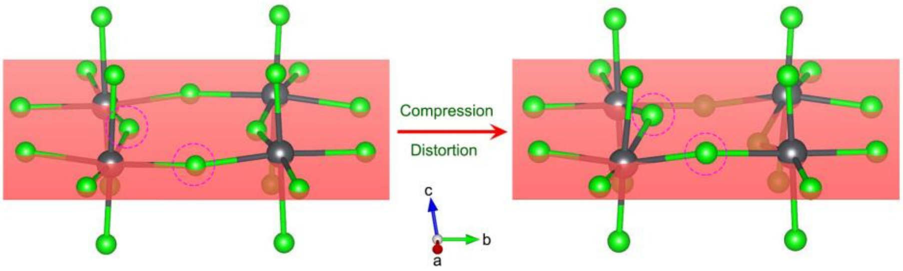

Figure S3. Schematic diagram of Pb-Br inorganic layer distortion in $(PEA)_2PbBr_4$ upon compression. Gray ball: Pb, green ball: Br.

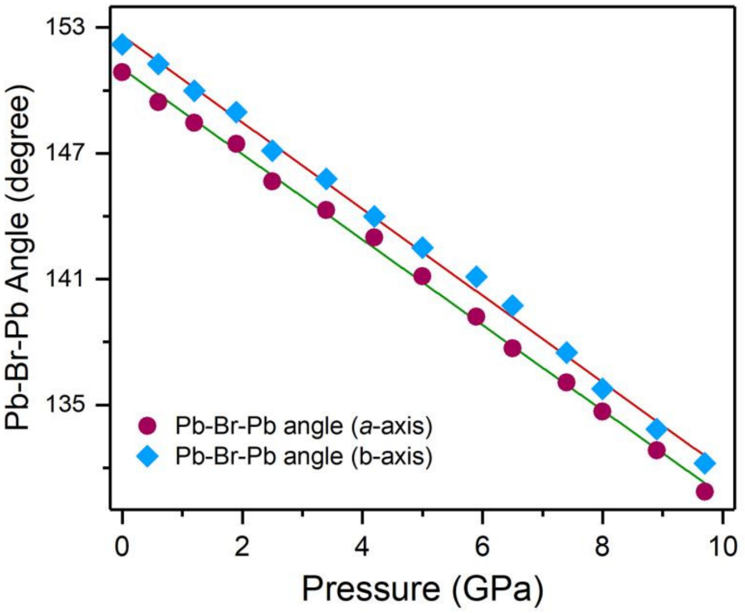

Figure S4. Rietveld refinement Pb-Br-Pb angle evolution in Pb-Br inorganic layer as a function of pressure.

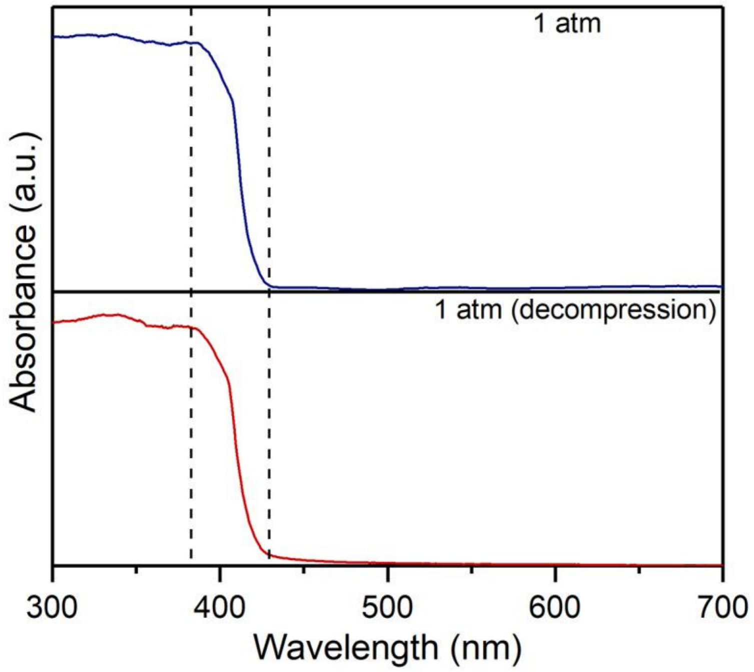

Figure S5. Absorption spectra of $(PEA)_2PbBr_4$ at ambient conditions and decompression.

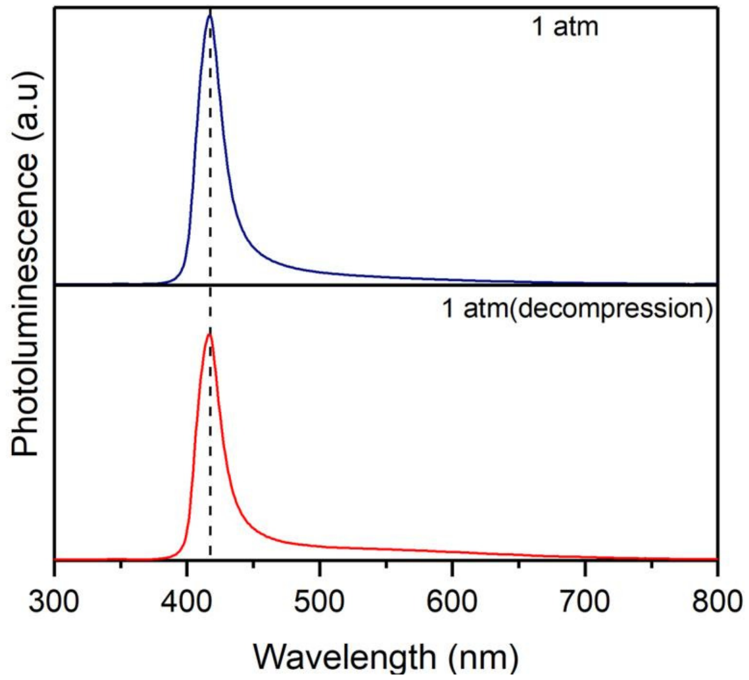

Figure S6. PL spectra of $(PEA)_2PbBr_4$ at ambient conditions and decompression.
